# Caregiver willingness to give TPT to children living with drug-resistant TB patients

**DOI:** 10.5588/ijtld.21.0760

**Published:** 2022-10-01

**Authors:** V. Rouzier, M. Murrill, S. Kim, L. Naini, J. Shenje, E. Mitchell, M. Raesi, M. Lourens, A. Mendoza, F. Conradie, N. Suryavanshi, M. Hughes, S. Shah, G. Churchyard, S. Swindells, A. Hesseling, A. Gupta, for the PHOENIX Feasibility Study team

**Affiliations:** 1 GHESKIO Centers, Port-au-Prince, Haiti and Weill Cornell Medicine, Center for Global Health, Department of Medicine, New York, NY; 2 Johns Hopkins Medical Institutions, Baltimore, MD; 3 Frontier Science Foundation, Brookline, MA; 4 Social and Scientific Systems, Inc., Silver Springs, MD, USA; 5 South African Tuberculosis Vaccine Initiative (SATVI), Cape Town, South Africa; 6 University of Cape Town Lung Institute, Mowbray, South Africa; 7 Gaborone Clinical Research Site, Gaborone, Botswana; 8 TASK Applied Science Clinical Research Site, Bellville, South Africa; 9 Asociación Civil Impacta Salud y Educación – Barranco Clinical Research Site, Lima, Peru; 10 Sizwe Tropical Disease Hospital, Johannesburg, South Africa; 11 Byramjee Jeejeebhoy Government Medical College Clinical Trials Unit, Pune, India; 12 Harvard T H Chan School of Public Health, Boston, MA; 13 Emory University Rollins School of Public Health, Atlanta, GA, USA; 14 School of Public Health, University of the Witwatersrand, Johannesburg, South Africa; 15 Aurum Institute, Johannesburg, South Africa; 16 University of Nebraska Medical Center, Omaha, NE, USA; 17 Desmond Tutu TB Centre, Stellenbosch University, Tygerberg, South Africa

**Keywords:** tuberculosis, contacts, pediatric, children, prophylaxis

## Abstract

**BACKGROUND:**

Pediatric household contacts (HHCs) of patients with multidrug-resistant TB (MDR-TB) are at high risk of infection and active disease. Evidence of caregiver willingness to give MDR-TB preventive therapy (TPT) to children is limited.

**METHODS:**

This was a cross-sectional study of HHCs of patients with MDR-TB to assess caregiver willingness to give TPT to children aged <13 years.

**RESULTS:**

Of 743 adult and adolescent HHCs, 299 reported caring for children aged <13 years of age. The median caregiver age was 35 years (IQR 27–48); 75% were women. Among caregivers, 89% were willing to give children MDR TPT. In unadjusted analyses, increased willingness was associated with TB-related knowledge (OR 5.1, 95% CI 2.3–11.3), belief that one can die of MDR-TB (OR 5.2, 95% CI 1.2–23.4), concern for MDR-TB transmission to child (OR 4.5, 95% CI 1.6–12.4), confidence in properly taking TPT (OR 4.5, 95% CI 1.6–12.6), comfort telling family about TPT (OR 5.5, 95% CI 2.1–14.3), and willingness to take TPT oneself (OR 35.1, 95% CI 11.0–112.8).

**CONCLUSIONS:**

A high percentage of caregivers living with MDR- or rifampicin-resistant TB patients were willing to give children a hypothetical MDR TPT. These results provide important evidence for the potential uptake of effective MDR TPT when implemented.

## Abstract (fr)

**CONTEXTE:**

Les enfants contacts familiaux (HHC) des patients atteints de TB multirésistante (MDR-TB) présentent un risque élevé d’infection et de maladie active. Les preuves de la volonté des soignants d’administrer un traitement préventif de la MDR-TB (TPT) aux enfants sont limitées.

**MÉTHODES:**

Il s’agit d’une étude transversale des centres de soins de santé de patients atteints de MDR-TB visant à évaluer la volonté des soignants d’administrer la TPT aux enfants âgés de <13 ans.

**RÉSULTATS:**

Sur 743 adultes et adolescents, 299 ont déclaré s’occuper d’enfants âgés de <13 ans. L’âge médian des soignants était de 35 ans (IQR 27–48) ; 75% étaient des femmes. Parmi les soignants, 89% étaient disposés à donner aux enfants des TPT MDR. Dans les analyses non ajustées, l’augmentation de la volonté était associée aux connaissances sur la TB (OR 5,1 ; IC 95% 2,3–11,3), la croyance que l’on peut mourir de la MDR-TB (OR 5,2 ; IC 95% 1,2–23,4), l’inquiétude concernant la transmission de la MDR-TB à l’enfant (OR 4.5 ; IC 95% 1,6–12,4), confiance dans la prise correcte du TPT (OR 4,5 ; IC 95% 1,6–12,6), aisance à parler du TPT à la famille (OR 5,5 ; IC 95% 2,1–14,3) et volonté de prendre le TPT soi-même (OR 35,1 ; IC 95% 11,0–112,8).

**CONCLUSIONS:**

Un pourcentage élevé de soignants vivant avec des patients atteints de MDR-TB ou TB résistante à la rifampicine étaient disposés à donner aux enfants un hypothétique TPT MDR. Ces résultats fournissent des preuves importantes de l’adoption potentielle d’un TPT MDR efficace lorsqu’il sera mis en œuvre.

Prior to COVID-19, TB was the primary infectious cause of mortality worldwide. Multidrug-resistant (MDR, resistant to at least isoniazid and rifampin [RIF]) and RIF-resistant (RR) TB were estimated to have caused 4.7% (465,000) of the 10 million new TB cases in 2019 and a disproportionate number of deaths.^1^ Household contacts (HHCs) of persons with infectious active TB are at high risk of infection and progression to active disease due to prolonged exposure in shared environments.^2^,^3^ This risk is highest in young children <5 years of age, where TB is particularly difficult to diagnose, and MDR/RR-TB treatment is lengthy with high toxicity rates.^4^,^5^ Treatment of latent infection is thus the cornerstone of pediatric MDR-TB prevention and an essential pillar of the End TB Strategy.^6^

Existing guidelines currently support the systematic testing and initiation of TB preventive therapy (TPT) for HHCs aged <5 years exposed to drug-susceptible TB.^7^ However, there are little data on specific treatment regimens for MDR-TB prevention in children.^8^ Diverse regimens of variable and unknown efficacy have been used for TPT,^9^–^21^ but data from randomized clinical trials remain unavailable to support regimens for MDR TPT in child HHCs. This critical gap is being addressed in three ongoing clinical trials: TB-CHAMP (Tuberculosis Child Multidrug-resistant Preventive Therapy Trial; ISRCTN92634082) and VQUIN (ACTRN12616000215426) evaluating 6 months of daily levofloxacin for prevention of MDR-TB in child HHCs, and PHOENIx (Protecting Households On Exposure to Newly Diagnosed Index Multidrug-Resistant Tuberculosis Patients) MDR-TB (NCT03568383) evaluating 6 months of delamanid for prevention of MDR-TB in high-risk HHCs, including children. There is, however, limited evidence on the uptake of TPT and the factors associated with uptake in families with children if an effective therapy were available.

Knowledge, attitudes, and practices (KAP) studies have the potential to provide insights into the willingness of individuals to utilize a proposed prevention strategy, as well as provide important context for implementation.^22^,^23^ We conducted a multi-country, cross-sectional study in diverse high TB burden settings of HHCs of MDR/RR-TB index cases, investigating the willingness of HHC caregivers to provide their children a hypothetical MDR TPT.

## METHODS

### Study setting

This study was conducted between October 2015 and April 2016 at 16 clinical research sites in eight countries: Botswana (1 site), Brazil (1), Haiti (1), India (2), Kenya (1), Peru (2), South Africa (7) and Thailand (1) in preparation for the PHOENIx trial being conducted by the AIDS Clinical Trials Group (ACTG; https://actgnetwork.org/) and International Maternal Pediatric Adolescent AIDS Clinical Trials Network (IMPAACT; http://impaactnetwork.org/). The study design has been discussed elsewhere^24^,^25^ and is summarized briefly below.

### Study participant eligibility criteria and recruitment

Index cases with pulmonary MDR/RR-TB were approached for enrollment if they met the following inclusion criteria: 1) documented RIF resistance using Xpert^®^ MTB/RIF (Cepheid, Sunnyvale, CA, USA), line-probe assay or phenotypic drug susceptibility testing; 2) initiated MDR-TB treatment within 6 months prior to study enrollment, 3) had at least one HHC, 4) provided permission to enumerate and screen HHCs, and 5) resided at a distance deemed by the site study team close enough for study conduct. HHCs were defined as any person 1) currently living or having lived in the same dwelling unit or plot of land, 2) sharing or having shared housekeeping arrangements with the index case, and 3) reporting exposure within 6 months prior to the index case starting MDR-TB treatment. A convenience sample of index cases was recruited from each clinical research site, and all HHCs were enumerated, screened for eligibility, and approached for enrollment. As previously reported, all HHCs aged ≥13 years were requested to complete a KAP questionnaire regarding MDR-TB.^24^ A subset of questions identified which HHCs were caring for children <13 years of age or a dependent of any age. The present analysis was restricted to HHCs who reported being responsible for a child <13 years of age or a dependent of any age. Because of ambiguous wording in the questionnaire, dependents were assumed to all be children in this analysis.

### Data collection and variables

A semi-structured knowledge, attitudes, and practices questionnaire was adapted for MDR-TB from a WHO guide for TB KAP survey development.^26^ Additional HHC information obtained included demographic, social, medical, and household characteristics. Questionnaires were administered in person by trained field staff or clinicians prior to participant education or counseling. The primary outcome in this analysis was caregiver’s willingness to have their children take a daily TPT pill to decrease their risk of MDR-TB. This outcome variable, as well as HHC caregiver willingness to have their children undergo a blood test (i.e., interferon-gamma release assay), provide a sputum sample, and have a chest radiograph were collected as categorical (yes, not sure, no) and dichotomized as ‘yes’ vs. ‘not sure’ or ‘no’ for analysis.

TB knowledge was analyzed as a binary variable, where “appropriate” knowledge was defined as correctly identifying all of the following: 1) cough ≥3 weeks is a symptom of TB; 2) TB is a curable disease; 3) TB is transmitted via air when an infected person coughs or sneezes; and 4) MDR-TB cure is possible through directly observed therapy.^27^ Confidence in ability to properly take MDR TPT was defined as HHCs feeling confident or very confident (5-point Likert scale) in being able to perform all five of the following: meeting with study staff monthly to take medications, coping with difficulties the medication may cause, taking all doses, continuing to take medications even if feeling healthy, and completing medications.

### Statistical analysis

The primary objective of this analysis was to characterize the willingness of caregivers to give their children daily MDR TPT, as well as to identify caregiver-level factors associated with willingness. Relevant caregiver-level factors evaluated were identified through literature review and informed by the Health Belief Model.^28^ Logistic regression models were fit using generalized estimating equations (GEE) to account for household-level clustering, but were unadjusted for clinical research site or other household contact-level factors due to inadequate outcome variability and insufficient sample size. Because of an ambiguity of wording in the questionnaire, sensitivity analyses were conducted to examine the robustness of unadjusted associations after restricting the analysis to households with an enumerated child <13 or <15 years of age. Additionally, the analysis was repeated after restricting it to households with at least one enumerated child <5 or 5–15 years of age to explore if factors associated with willingness to give TPT were similar for different age ranges of child HHCs. All analyses were conducted in Stata v13.1 (StataCorp, College Station, TX, USA), except for the creation of stacked bar charts, which were created in R v4.0.2 (R Computing, Vienna, Austria) using the *likert* package.^29^

### Human research ethics approvals

Ethical approval was obtained from each research site’s local institutional review board. Written informed consent was obtained for all participating MDR-TB index cases and their household contacts prior to study interviews and procedures.

## RESULTS

### Study recruitment and eligibility

Across all sites, 328 adult pulmonary MDR-TB/RR-TB index cases were screened during the recruitment period; 7 declined screening and 13 were ineligible. Three declined contact with HHCs, and 27 had no eligible, enrolled HHCs who also completed the KAP questionnaire. The final study sample included 278 index cases and 743 HHCs who completed the KAP questionnaire. The median number of eligible, enumerated HHCs of all ages in these index case households was 4 (interquartile range [IQR] 2–5), with 32% of households having at least one adolescent HHC (aged 13–17 years), 43% at least one child aged 5–12 years, and 35% at least one child aged <5 years. Of the 743 HHCs, 299 (40.2%) reported being responsible for the care of a child less than 13 years or dependent of any age.

### Characteristics of MDR/RR-TB HHCs who are caregivers

The majority of HHCs who reported being caregivers were women (74.9%). The median age of caregiver HHCs was 35 years (IQR 27–48), 60.2% had completed at least secondary education, and 39.8% were currently employed or in school. Prior TB treatment was reported by 38 (12.7%) caregivers and current tobacco use by 65 (21.7%). Almost daily alcohol use at any point in the past year was reported by 22 caregivers (7.4%). Other substance use (e.g., marijuana, cocaine) at any point in the past year was reported by 20 (6.7%) HHCs (Table).

**Table:** Characteristics of MDR-TB household contacts who are caregivers (n = 299) and unadjusted associations between caregiver factors and their willingness in having their children take MDR-TB preventive therapy

Caregiver/guardian variables	Caregivers *N* = 299 *n* (%)	Willingness to have children take TPT *n/N* (%)*	OR^†^ (95% CI)	*P* value
Sociodemographic characteristics				
Age, years, median [IQR]^‡^	35 [27–48]	—	0.98 (0.95–1.01)	0.261
Sex				
Female	224 (74.9)	210/224 (93.8)	1.50 (0.63–3.60)	0.364
Male	75 (25.1)	68/74 (90.7)	1.0 (reference)	
Education level				
Secondary education or greater	180 (60.2)	168/180 (93.3)	1.36 (0.49–3.75)	0.550
None or primary school	119 (39.8)	110/119 (92.4)	1.0 (reference)	
Currently employed or in school				
Yes	119 (39.8)	109/119 (91.6)	0.77 (0.37–1.62)	0.497
No (reference)	180 (60.2)	169/180 (93.9)	1.0 (reference)	
Perceived susceptibility to and severity of MDR-TB				
TB-related knowledge^§^				
Appropriate	232 (77.6)	223/232 (96.1)	5.06 (2.26–11.31)^¶^	<0.001^¶^
Incomplete	67 (22.4)	55/67 (82.1)	1.0 (reference)	
Can die of MDR-TB without treatment				
Yes	280 (93.7)	264/280 (94.3)	5.19 (1.15–23.41)^¶^	0.032^¶^
No/don’t know	19 (6.4)	14/19 (73.7)	1.0 (reference)	
Concerned about child getting MDR-TB from index case				
Yes	238 (79.6)	201/208 (96.6)	4.45 (1.60–12.41)^¶^	0.004^¶^
No/neutral	61 (20.4)	77/91 (84.6)	1.0 (reference)	
Belief that TB is a serious problem in community				
Yes	204 (68.2)	194/204 (95.1)	2.39 (0.95–5.98)	0.064
No/neutral	95 (31.8)	84/95 (88.4)	1.0 (reference)	
Perception of how person with TB in community is treated				
Most people in community reject person with TB	68 (22.7)	61/68 (89.7)	0.89 (0.30–2.62)	0.830
Most people are friendly but avoid/mostly supportive	231 (77.3)	217/231 (93.9)	1.0 (reference)	
Barriers and enablers to preventive therapy				
Comfortable telling family about preventive therapy				
Yes	217 (72.6)	210/217 (96.8)	5.47 (2.09–14.32)^¶^	0.001^¶^
No/neutral	82 (27.4)	68/82 (82.9)	1.0 (reference)	
Confident in ability to properly take preventive therapy^#^				
Yes	188 (62.9)	182/188 (96.8)	4.53 (1.63–12.62)^¶^	0.004^¶^
No/neutral	111 (37.1)	96/111 (86.5)	1.0 (reference)	
Willing to obtain all prerequisite tests				
Yes	283 (94.7)	268/283 (94.7)	10.23 (2.73–38.29)^¶^	0.001^¶^
No	16 (5.4)	10/16 (62.5)	1.0 (reference)	
Willing to take preventive therapy self				
Yes	266 (89.0)	260/266 (97.7)	35.14 (10.95–112.75)^¶^	<0.001^¶^
No/not sure	33 (11.0)	18/33 (54.6)	1.0 (reference)	
Medical and social history				
Any current TB-related symptoms				
Yes	60 (20.1)	54/60 (90.0)	0.70 (0.28–1.77)	0.453
No	239 (79.9)	224/239 (93.7)	1.0 (reference)	
Alcohol use in past 12 months				
Yes	22 (7.4)	21/22 (95.5)	1.42 (0.48–4.24)	0.529
No/refused to answer	277 (92.6)	257/277 (92.8)	1.0 (reference)	
Drug use: ever used in past 12 months				
Yes	20 (6.7)	19/20 (95.0)	1.32 (0.36–4.79)	0.676
No/refused to answer	279 (93.3)	259/279 (92.8)	1.0 (reference)	
Previously treated for TB				
Yes	38 (12.7)	36/38 (94.7)	1.22 (0.36–4.13)	0.754
No/unknown	261 (87.3)	242/261 (92.7)	1.0 (reference)	

* Number of caregivers willing to give children MDR-TB preventive therapy/total number of caregivers.

† Logistic models fit using GEE to account for household-level clustering; unadjusted for clinical research site or other household contact-level factors.

‡ OR is per year of age.

§ Binary variable where “appropriate” knowledge was defined as correctly answering 4 questions about TB symptoms, transmission, treatment; incomplete was defined as incorrectly answering ≥1 question.

¶ Statistically significant.

# Confidence in ability to properly take TPT: defined as feeling confident or very confident (5-point Likert scale) in being able to perform all 5 of the following: meeting monthly to take medications, coping with medication difficulties, taking all doses, continuing to take medications even if feeling healthy, and completing medications.

MDR-TB = multidrug-resistant TB; TPT = TB preventive therapy; OR = odds ratio, CI = confidence interval; IQR = interquartile range; GEE = generalised estimating equations.

### Caregiver willingness in having their children take daily MDR TPT

Overall, 278 (92.9%) of caregivers were willing to give MDR TPT to children. This willingness was high at all sites (IQR 79–100%), except for one site in India (0%), where only a single HHC caregiver was interviewed who was unsure about giving MDR TPT to their children (Figure). Nearly all (94.7%) caregivers were willing to have children complete prerequisite steps to determine MDR TPT eligibility, including blood testing, providing a sputum sample and obtaining a chest radiograph. In unadjusted analysis, increased willingness to give TPT to children was associated with HHC-level factors related to perceived susceptibility to MDR-TB, belief that MDR-TB is a severe condition, as well as enablers to taking therapy. The strongest positive associations with higher willingness were TB-related knowledge (odds ratio [OR] 5.1, 95% confidence interval [CI] 2.3–11.3), belief that one can die of MDR-TB without treatment (OR 5.2, 95% CI 1.2–23.4), concern about child getting MDR-TB from index case (OR 4.5, 95% CI 1.6–12.4), confidence in ability to properly take MDR TPT (OR 4.5, 95% CI 1.6–12.6), being comfortable telling others about taking MDR TPT (OR 5.5, 95% CI 2.1–14.3) and caregivers’ willingness to take TPT (OR 35.14, 95% CI 10.95–112.75). In sensitivity analyses, caregiver willingness to give children MDR TPT remained high and factors associated with this willingness were highly similar after restricting the analytic population to households with at least one child aged <5(*n* = 176), 5–15 (*n* = 224), <13 (*n* = 268) or ≤15 years (*n* = 279) (Supplementary Table S1). In secondary analyses, caregivers also reported high levels of willingness to enroll their children in a study evaluating a new medicine to protect against MDR-TB; 185 of 246 (75.2%) caregivers of very young children less than 5 were willing or very willing (Supplementary Figure S1).

**Figure:**
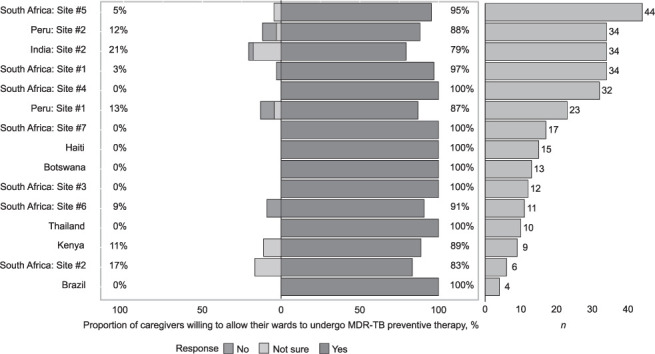
MDR/RR-TB household contacts who are caregivers of children (n = 299) and their willingness to give MDR-TB preventive therapy by clinical research site (left panel); number of enrolled caregivers at each clinical research site (right panel). India site #1 data not shown: n = 1 caregiver. MDR-TB = multidrug-resistant TB; RR-TB = rifampicin-resistant TB.

## DISCUSSION

Child contacts, especially those who are under the age of 5 years, are at high risk for developing MDR-TB disease after exposure. Current MDR TPT recommendations^29^ are based on limited observational evidence^30^ and expert opinion, filling the gap while awaiting the results of three ongoing randomized clinical trials evaluating the efficacy of levofloxacin or delamanid. In this large multi-center study of MDR/RR-TB household contacts, 92% of caregivers were willing to give MDR TPT to their children, and 94% were willing to have them complete prerequisite evaluations. Given the high willingness of caregivers to give MDR TPT, the present study provides evidence to support the uptake of MDR TPT among child HHCs who are at high risk of infection and progression to MDR-TB disease. The strongest factors associated with increased willingness were related to willingness to take TPT oneself, concerns about transmission and the fatality risk of disease, knowledge of TB, confidence in ability to properly take TPT, as well as comfort in telling family members about TPT. These findings can inform MDR TPT counseling efforts or offer starting points for subsequent studies.

The potential uptake of MDR TPT in the present study was similar to high proportions (>80%) of MDR TPT initiation observed in prior observational studies (Supplementary Table S1) from South Africa, United States, and Micronesia among eligible children contacts in different outbreak settings (school, daycare center and households).^9^,^10^,^13^,^17^ In these prior studies, reported reasons for not starting TPT were limited, but included parent refusal and the absence of an adult who could provide consent. To note, children who initiated MDR TPT regimens had low rates of side effects and high rates of treatment completion (>90%), particularly for regimens not containing pyrazinamide.^30^,^31^

The strengths of the present study are notably the inclusion of multiple diverse study settings, a focus on a key population in efforts to prevent MDR-TB and a directly policy relevant research question on potential uptake of MDR TPT. The primary outcome of self-reported willingness to give MDR TPT to children, however, may not be a strong proxy for completion of treatment. While the focus of this study was the willingness of caregivers to have child HHCs take MDR TPT, the wording of the primary outcome question was ambiguous in including “dependents regardless of age.” Results were, however, robust to restricting the analytic population to households that had at least one enumerated child HHC <13 years of age. Results were also robust to restricting the analytic population to households with at least one enumerated child <5 or 5–15 years of age. Assuming caregivers who completed the KAP questionnaire were responsible for all enumerated children in a household, these results suggest that factors associated with willingness to give MDR TPT to child HHCs are similar for children of different age ranges. Another potential limitation is the wording of TPT, which was phrased as being a one-pill daily regimen, which could positively have influenced caregivers’ acceptance and response. A high pill burden regimen might be less well received and affect caregivers’ willingness to provide TPT to their children. As discussed previously,^21^ there is also the possibility of selection bias in that HHCs of eligible index cases who had initiated TB treatment may be different from HHCs of TB patients who had not initiated treatment, and we had insufficient sample size to adjust for relevant covariates in the analysis.

In conclusion, the high willingness of caregivers to administer a theoretical newly developed MDR TPT to their children provides important and timely support for the probability of successful uptake of an effective MDR TPT when recommended and implemented. Strategies to enhance uptake of TPT and address barriers to completion will be essential to scale up of recommendations for child HHC TPT. The identified predictors of uptake of TPT for children highlight the need for education and counseling efforts on appropriate TB knowledge targeting female caregivers in particular, who most commonly bring small children in for health services. Limited understanding of TB-related mortality and potential concern over TB-related stigma in the community were associated with a decreased willingness to provide IPT to young children, emphasizing the importance of community-based education and support strategies. Further research to evaluate strategies to support roll out of TPT and especially help families achieve high completion rates will be crucial to reduce the burden of MDR-TB disease in children.
