# Supplementary material for: Caregiver willingness to give TPT to children living with drug-resistant TB patients
Source: Int J Tuberc Lung Dis. 2022 Oct 1;26(10):949–55. doi: 10.5588/ijtld.21.0760 (PMC9524515; doi:10.5588/ijtld.21.0760)
Supplement: Supplementary file 1 [file iutld_ijtld_21.0760_supplementarydata1.pdf]

http://dx.doi.org/10.5588/ijtld.21.0760

Caregiver willingness to give TPT to children living with drugresistant TB patients

Supplementary S1 Figure: Flow Diagram of patients approached for study participation

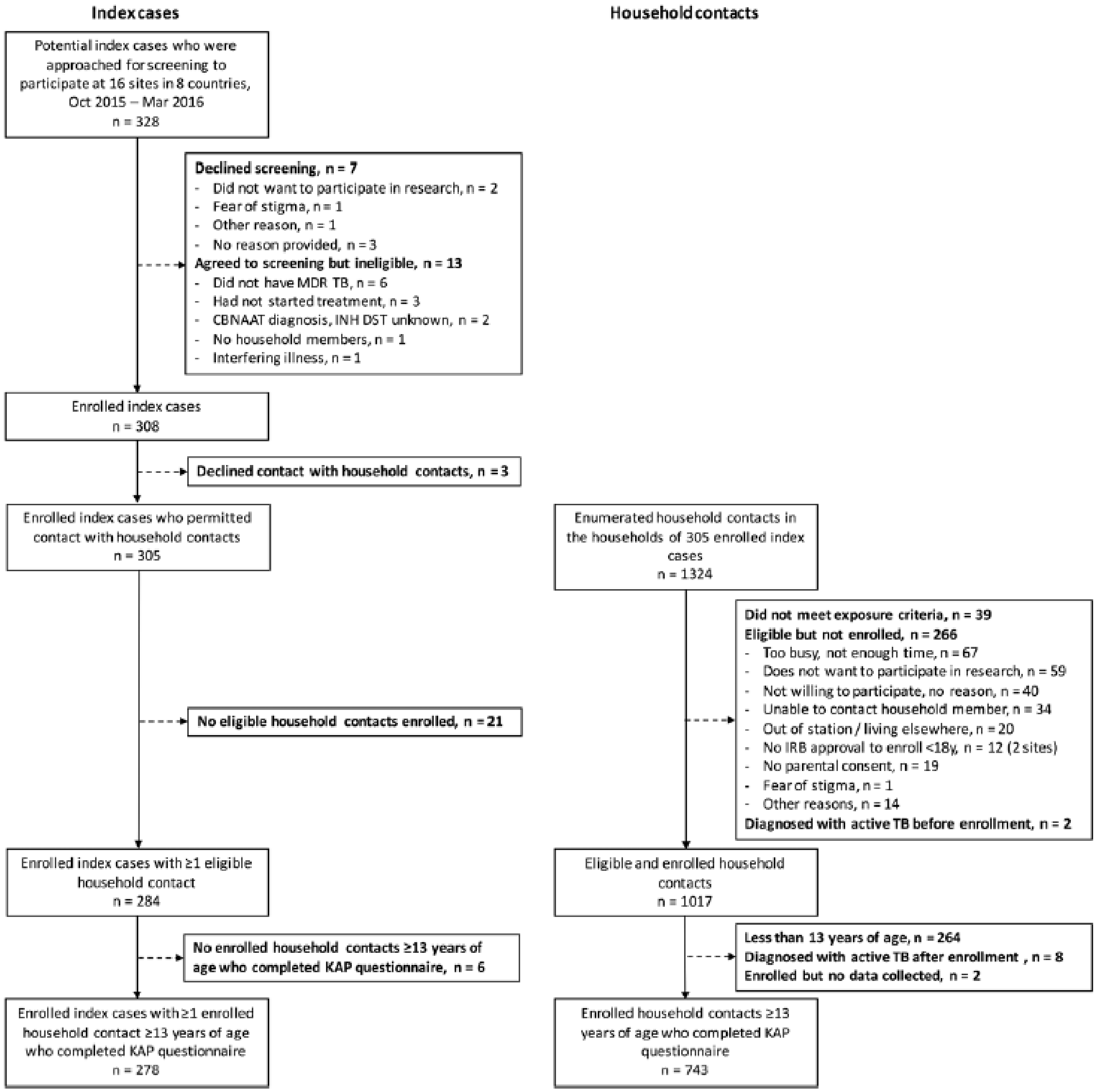

Supplementary Table S1: Sensitivity analyses of unadjusted associations between caregiver factors and their willingness to give their dependents a newly developed MDR TB preventive therapy

Covariates	All caregivers (n = 299)			Sensitivity Analyses: Household Contact <5 years of age						Sensitivity Analyses: Household Contact 5-15 years of age					
				Restrict to ≥1 enumerated HHC <5y (n = 176) Data Source: HH Enumeration			Restrict to reported HHC <5y (n = 159) Data source: KAP Questionnaire			Restrict to ≥1 enumerated HHC 5-15y (n = 224) Data Source: HH Enumeration			Restrict to reported HHC 5-15y (n = 228) Data source: KAP Questionnaire		
	OR	95%CI	p	OR	95%CI	p	OR	95%CI	p	OR	95%CI	p	OR	95%CI	p
**Sociodemographic Characteristics**															
Age*	0.98	[0.95,1.01]	0.261	0.99	[0.92,1.08]	0.900	1.00	[0.92,1.08]	0.982	0.98	[0.95,1.01]	0.221	0.98	[0.94,1.01]	0.220
Female gender (Ref: male)	1.50	[0.63,3.60]	0.364	0.52	[0.10,2.85]	0.452	0.56	[0.10,3.25]	0.515	1.52	[0.63,3.67]	0.347	1.49	[0.61,3.66]	0.386
Secondary education or greater (Ref: none / primary)	1.36	[0.49,3.75]	0.550	2.31	[0.52,10.27]	0.270	2.30	[0.52,10.14]	0.271	1.30	[0.49,3.49]	0.598	0.96	[0.33,2.81]	0.938
Currently employed or in school (Ref: no)	0.77	[0.37,1.62]	0.497	1.85	[0.37,9.15]	0.451	2.00	[0.40,9.94]	0.398	0.83	[0.40,1.69]	0.605	0.77	[0.36,1.65]	0.507
**Perceived Susceptibility to and Severity of MDR TB**															
Appropriate TB knowledge (Ref: incomplete)	**5.06**	**[2.26,11.31]**	**<0.001**	**8.75**	**[2.71,28.28]**	**<0.001**	**5.81**	**[1.82,18.54]**	**0.003**	**4.79**	**[2.12,10.82]**	**<0.001**	**4.94**	**[2.14,11.43]**	**<0.001**
Can die of MDR TB without treatment (Ref: no / don't know)	**5.19**	**[1.15,23.41]**	**0.032**	--	--	--	--	--	--	**6.74**	**[1.39,32.65]**	**0.018**	**8.31**	**[1.62,42.67]**	**0.011**
Concerned about child getting MDR-TB from index case (Ref: no / neutral)	**4.45**	**[1.60,12.41]**	**0.004**	4.15	[0.89,19.23]	0.069	4.49	[0.97,20.74]	0.054	**4.24**	**[1.50,11.99]**	**0.006**	**3.29**	**[1.13,9.57]**	**0.029**
Belief that TB is a serious problem in community (Ref: no / neutral)	2.39	[0.95,5.98]	0.064	3.57	[0.85,14.93]	0.082	3.83	[0.91,16.08]	0.066	2.39	[0.94,6.05]	0.066	2.25	[0.88,5.77]	0.090
Most people in community reject person with TB (Ref: other)	0.89	[0.30,2.62]	0.830	0.31	[0.07,1.42]	0.132	0.38	[0.08,1.75]	0.215	0.93	[0.33,2.69]	0.900	1.04	[0.32,3.37]	0.941
**Barriers and Enablers to Preventive Therapy**															
Comfortable telling family about PT (Ref: no / neutral)	**5.47**	**[2.09,14.32]**	**0.001**	3.80	[0.91,15.97]	0.068	**6.78**	**[1.56,29.52]**	**0.011**	**5.29**	**[2.02,13.89]**	**0.001**	**5.44**	**[2.06,14.38]**	**0.001**
Confident in properly taking preventive therapy (Ref: no / neutral)	**4.53**	**[1.63,12.62]**	**0.004**	**14.21**	**[1.54,131.54]**	**0.019**	5.54	[0.94,32.59]	0.058	**4.35**	**[1.61,11.71]**	**0.004**	**5.34**	**[2.01,14.18]**	**0.001**
Willing to obtain all prerequisite tests (Ref: no)	**10.23**	**[2.73,38.29]**	**0.001**	**11.05**	**[1.86,65.78]**	**0.008**	7.91	[0.99,63.02]	0.051	**12.08**	**[2.87,50.78]**	**0.001**	**26.24**	**[6.08,113.25]**	**<0.001**
Willing to take preventive therapy self (Ref: no / not sure)	**35.14**	**[10.95,112.75]**	**<0.001**	**23.90**	**[4.73,120.61]**	**<0.001**	**19.60**	**[3.88,98.93]**	**<0.001**	**32.42**	**[9.69,108.46]**	**<0.001**	**39.53**	**[10.91,143.29]**	**<0.001**
**Medical and Social History**															
Any current TB-related symptoms (Ref: no)	0.70	[0.28,1.77]	0.453	0.68	[0.17,2.72]	0.590	0.59	[0.15,2.35]	0.451	0.70	[0.29,1.70]	0.428	0.65	[0.25,1.70]	0.377
Alcohol use in past 12m (Ref: no / refused to answer)	1.42	[0.48,4.24]	0.529	--	--	--	--	--	--	1.56	[0.51,4.76]	0.438	1.35	[0.45,4.04]	0.593
Drug use: ever used in past 12m (Ref: no / refused to answer)	1.32	[0.36,4.79]	0.676	--	--	--	--	--	--	1.49	[0.43,5.21]	0.529	1.46	[0.44,4.77]	0.535
Previously treated for TB (Ref: no / unknown)	1.22	[0.36,4.13]	0.754	--	--	--	--	--	--	1.26	[0.38,4.17]	0.704	1.20	[0.36,3.96]	0.764

*Age = continuous variable

-- = inadequate sample size for analysis
